# Improving Stem Cell Delivery to the Trabecular Meshwork Using Magnetic Nanoparticles

**DOI:** 10.1038/s41598-018-30834-7

**Published:** 2018-08-16

**Authors:** E. J. Snider, K. P. Kubelick, K. Tweed, R. K. Kim, Y. Li, K. Gao, A. T. Read, S. Emelianov, C. R. Ethier

**Affiliations:** 1 Wallace H. Coulter Department of Biomedical Engineering, Georgia Institute of Technology and Emory University, Atlanta, Georgia; 2 School of Electrical and Computer Engineering, Georgia Institute of Technology, Atlanta, Georgia

**Keywords:** Mesenchymal stem cells, Glaucoma

## Abstract

Glaucoma is a major cause of blindness and is frequently associated with elevated intraocular pressure. The trabecular meshwork (TM), the tissue that primarily regulates intraocular pressure, is known to have reduced cellularity in glaucoma. Thus, stem cells, if properly delivered to the TM, may offer a novel therapeutic option for intraocular pressure control in glaucoma patients. For this purpose, targeted delivery of stem cells to the TM is desired. Here, we used magnetic nanoparticles (Prussian blue nanocubes [PBNCs]) to label mesenchymal stem cells and to magnetically steer them to the TM following injection into the eye’s anterior chamber. PBNC-labeled stem cells showed increased delivery to the TM vs. unlabeled cells after only 15-minute exposure to a magnetic field. Further, PBNC-labeled mesenchymal stem cells could be delivered to the entire circumference of the TM, which was not possible without magnetic steering. PBNCs did not affect mesenchymal stem cell viability or multipotency. We conclude that this labeling approach allows for targeted, relatively high-efficiency delivery of stem cells to the TM in clinically translatable time-scales, which are necessary steps towards regenerative medicine therapies for control of ocular hypertension in glaucoma patients.

## Introduction

Glaucoma is a leading cause of blindness, affecting over 70 million people worldwide. Its incidence is expected to continue to rise as the population ages^1,2^. The best-established risk factor for glaucoma is elevated intraocular pressure (IOP), which is determined by the rate of aqueous humor production within the eye and the subsequent outflow of aqueous humor through drainage pathways in the anterior eye^3^. The primary aqueous humor drainage route is known as the conventional pathway, consisting of the trabecular meshwork (TM), Schlemm’s canal, and, eventually, the circulatory system^4,5^.

In glaucoma patients, the cellularity of the TM is reduced^6–9^. The TM, along with the inner wall of Schlemm’s canal, is known to be the major site of outflow resistance. Further, TM cells phagocytose debris from aqueous humor to prevent outflow blockage and are contractile, which can change outflow resistance^10–12^. Thus, reduced TM cellularity presumably leads to tissue dysfunction and subsequent increased outflow resistance and elevated IOP. Therapies focused on restoring TM cellularity and function could therefore offer a therapeutic benefit to glaucoma patients with elevated IOP.

Towards this end, regenerative medicine therapies are being developed for the glaucomatous TM. Most existing studies have used fibroblast-derived induced pluripotent stem cells (iPSCs) or mesenchymal stem cells (MSCs)^13–17^. While each of these studies show the potential for regenerating the TM, delivery of cells after injection into the anterior chamber has relied on the stem cells being passively carried by aqueous humor outflow to the TM. Passive delivery of stem cells has led to inconsistent MSC delivery to the TM and non-specific delivery to other anterior ocular tissues^16,17^. Cell delivery to sites other than the TM is undesirable since stem cells may differentiate to undesired phenotypes in tissues such as the lens or cornea.

Passive delivery is further complicated by aqueous humor outflow dynamics. Outflow from the TM is known to be non-uniform (segmental) around the TM circumference^18^. Indeed, studies indicate that as little as one-third of the TM is actively filtering at any one time^19^, suggesting that passively delivered stem cells could integrate into as little as one-third of the TM. Such an outcome is presumably sub-optimal; for example, it is possible that active filtering regions may change over time (see e.g. work of [Braakman *et al*., 2015], [Pedrigi *et al*., 2011] on dynamic pores in the inner wall of Schlemm’s canal), although we do not have any direct evidence of such changes over the time scales of months to years that are relevant to human glaucoma^20,21^. Further, outflow is more segmental in glaucomatous patients than in unaffected individuals^22,23^, so that this problem is even worse in the target patient population.

To improve on conventional (passive) delivery, we propose to use magnetic nanoparticles to label MSCs and steer them to the TM using a magnetic field. Superparamagnetic iron oxide nanoparticles (SPIONs) have previously been used for targeted cell delivery to the retina^24,25^. For this study, we use Prussian blue nanocubes (PBNCs), which combine Prussian blue pigment with SPIONs^26^. The SPION core gives PBNCs magnetic properties that allow magnetic steering, while the Prussian blue pigment makes the particles strong absorbers of near-infrared light to allow minimally invasive imaging. We recently demonstrated that ultrasound-photoacoustic non-invasive imaging can visualize anterior segment tissues, as well as the delivery of cells labeled with PBNCs^27^. Here, we ask the question, are PBNCs suitable for MSC labeling and can they improve MSC delivery to the TM?

## Results

### MSCs Uptake PBNCs

**Figure 1:**
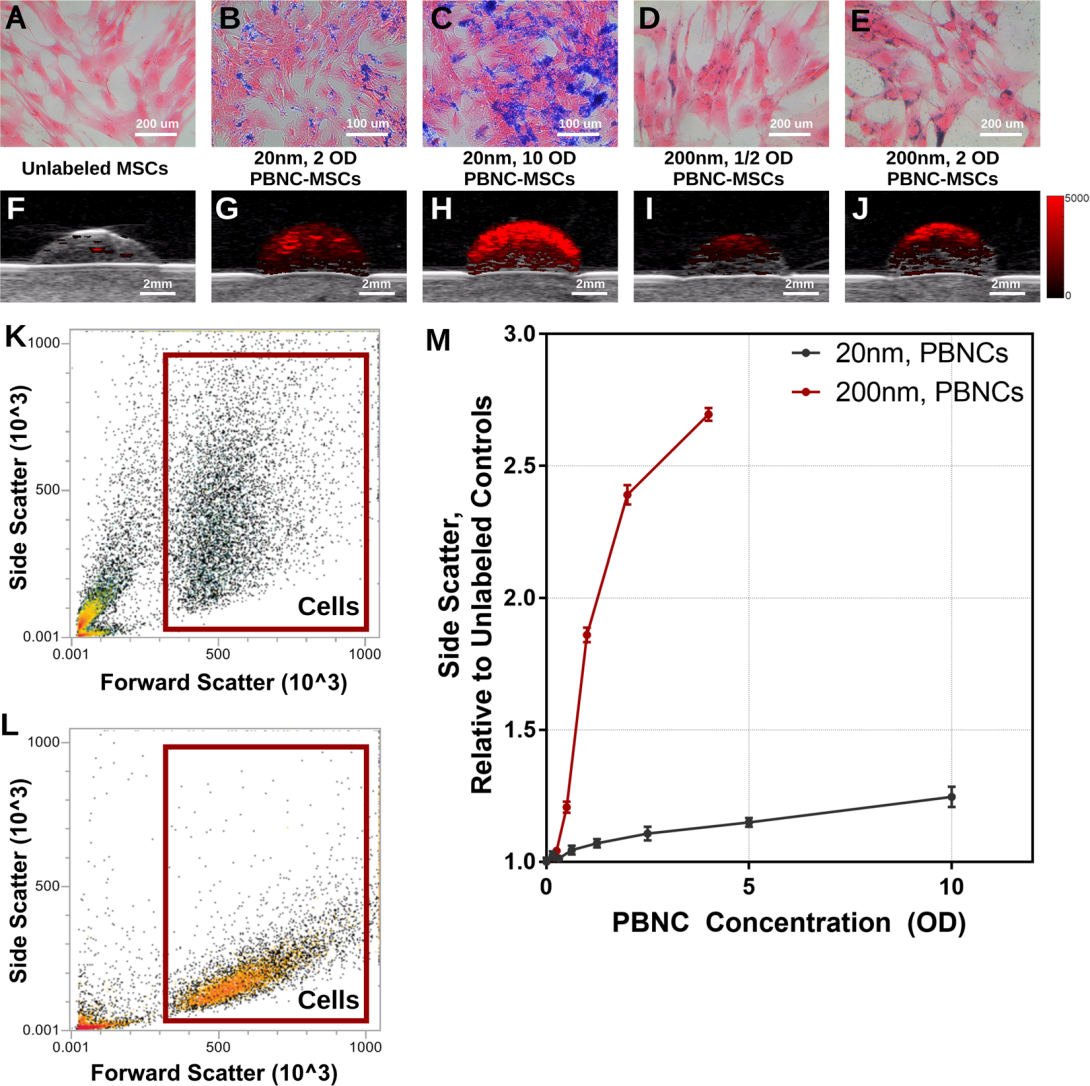
MSCs Take Up PBNCs. **(A–E)** Representative micrographs (from n = 3 technical replicates of 2 MSC donor strains) of MSCs after incubation with different sizes of PBNCs and in PBNC solutions of different concentrations, as quantified by solution optical density (OD). MSCs were eosin stained to visualize cytoplasm (pink), while PBNCs appeared blue. **(F–J)** Photoacoustic signal using 690 nm (20 nm PBNCs) or 755 nm (200 nm PBNCs) wavelength, visualizing labelled MSCs in gelatin phantoms. For unlabeled MSCs, signals were minimal for both 690 nm and 755 nm wavelengths, so only the image from 690 nm is shown. **(K,L)** Uptake of PBNCs was further assessed by light side scatter (SSC). Representative forward scatter (FSC) vs. SSC density maps collected by flow cytometry for **(K)** MSCs incubated in a solution of 200 nm PBNCs at a concentration of 2 OD or **(L)** unlabeled MSCs. Gating used for selecting MSCs is shown. **(M)** Normalized side scatter vs. optical density of PBNC incubating solution for 20 nm and 200 nm PBNC-MSCs (n = 3 technical replicates for each). Error bars denote standard deviation.

MSCs were incubated overnight in a solution containing either 20 nm or 200 nm PBNCs (Supplementary Fig. 1), after which cells were fixed and assessed for PBNC uptake by light microscopy. Prussian blue labeling was visible and colocalized with eosin-stained MSCs after incubation with either 20 nm or 200 nm PBNCs (Fig. 1A–E). PBNCs are known photoacoustic (PA) absorbers^26^, so PA imaging of labeled MSCs was performed by suspending cells in gelatin phantoms and imaging at 690 or 755 nm wavelengths for 20 nm or 200 nm PBNCs, respectively. PA signal was detectable and increased with the optical density (OD) of the PBNC solution used during cell labeling, i.e. with increasing PBNC concentration (Fig. 1F–J). PBNC uptake was further confirmed by flow cytometry. Specifically, side scatter, a measure of light scatter due to particles inside MSCs (i.e. nucleus, endosomes, PBNCs), increased following labeling with PBNCs when compared to unlabeled controls (Fig. 1K,L), with increases correlating with PBNC concentration. 200 nm PBNCs produced more side scattering than 20 nm PBNCs (Fig. 1M), consistent with scattering theory in which particles much smaller than the wavelength of light produce less side scatter than larger particles^28,29^. Overall, we conclude that both 20 nm and 200 nm PBNCs can label MSCs.

### PBNC-labeled MSCs can be steered by magnetic fields

**Figure 2:**
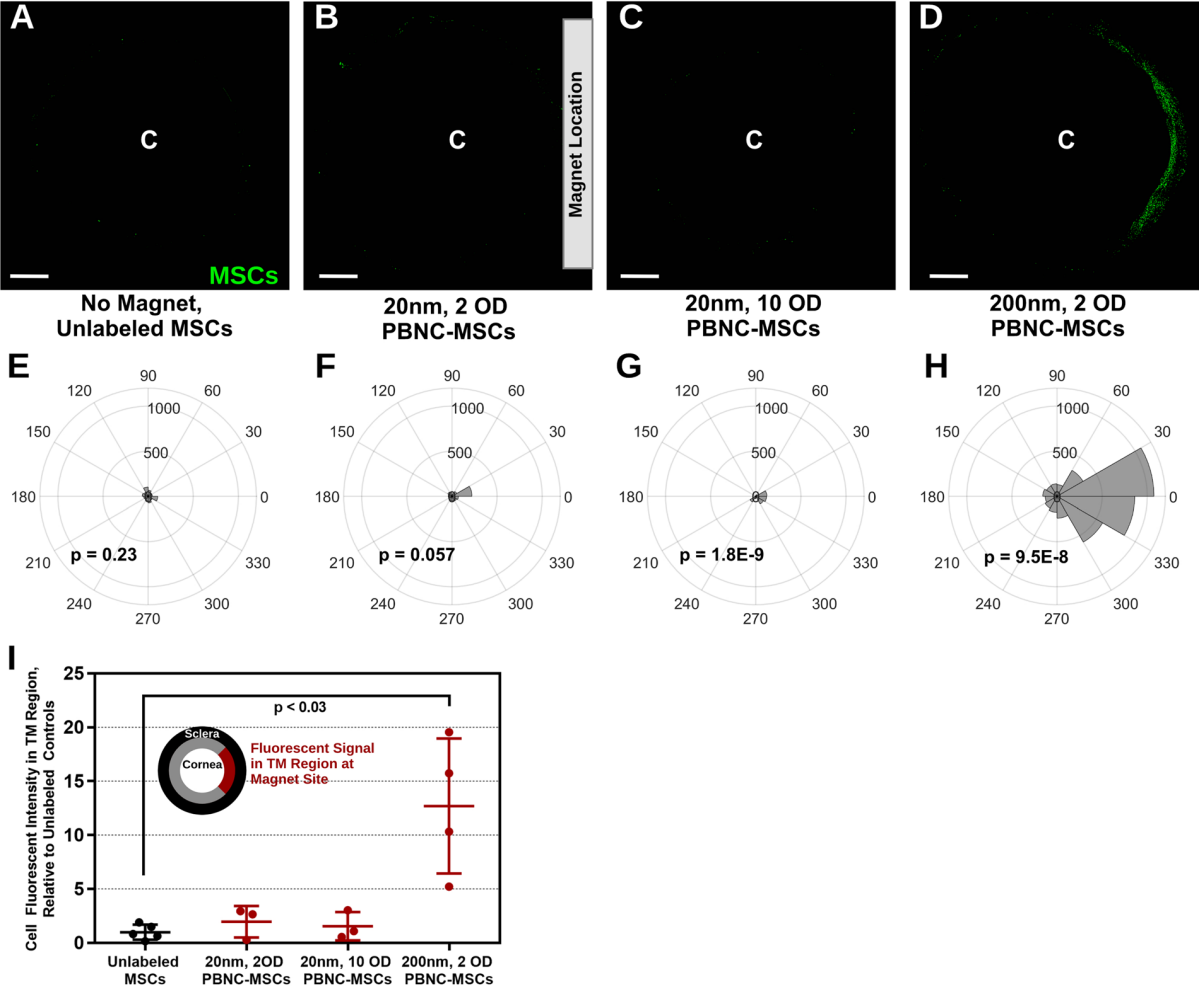
Magnetic Steering of MSCs labeled with either 20 or 200 nm PBNCs. **(A–D)** Representative *en face* micrographs of the anterior region of the eye after MSC delivery. Bar magnets, diagramed in (B) (to scale), were placed near the limbal region overnight in PBNC-MSC injected eyes. Cells that were steered to the TM appear as a green arc. “C” denotes the approximate center of the cornea. Note that the image has been masked so that only signal from the putative TM region is shown (see Methods). Scale bars denote 2 mm **(E–H)** The polar histograms illustrate the total fluorescence intensity within the TM region (plotted on the radial coordinate, in arbitrary units) for 30° sectors around the eye. Note that a bar magnet was placed adjacent to the limbus at 0° overnight. p-values were calculated using Kuiper’s V test to assess whether the distribution was non-uniformly skewed towards the center of the magnet location (0°). **(A,E)** no magnet, unlabeled MSCs (n = 5 eyes), **(B,F)** 20 nm, 2 OD PBNC-MSCs (n = 3 eyes), **(C,G)** 20 nm, 10 OD PBNC-MSCs (n = 3 eyes), and **(D,H)** 200 nm, 2 OD PBNC-MSCs (n = 4 eyes) experiments are shown. **(I)** Quantification of total fluorescent signal in the TM adjacent to the magnet (defined as the wedge extending from 45° to −45°) relative to total fluorescent signal adjacent to the magnet from eyes injected with MSCs lacking PBNC labeling. Individual data points are shown with central bars indicating mean values and error bars denoting standard deviation. Significance (p < 0.05) was determined by Kruskal-Wallis test, with Dunn’s post hoc analysis.

Either 20 or 200 nm PBNCs were used to label MSCs (hereinafter referred to as PBNC-MSCs), as described above. Cells were also labelled with a fluorescent tag, so that their distribution within the eye could be assessed. 250,000 labeled cells were injected into perfused porcine anterior segments maintained in organ culture. Perfused anterior segments are a widely-used *ex vivo* model for studying aqueous humor dynamics^30,31^ (Supplementary Fig. 2), and were used to determine the effectiveness of different approaches for steering injected cells to the TM. To establish a baseline for comparison, unlabeled MSCs were injected without an external magnet, so that MSC transport to the TM relied on normal fluid flow patterns towards the TM. This approach resulted in very few cells in the TM region (Fig. 2A). It was expected that cells would be attracted to a neodymium rectangular magnet placed near the limbus in one quadrant of the anterior segment (approximately 40mT field strength at center of eye, as determined by a Gaussmeter). However, when MSCs were labeled with 20 nm PBNCs in a solution with a concentration of 2 OD, few cells preferentially accumulated in the quadrant adjacent to the magnet (Fig. 2B). On the other hand, when the PBNC concentration in the incubation solution was increased to 10 OD, more MSC accumulation was detected near the magnet (Fig. 2C). Finally, if cells were incubated with 200 nm PBNCs at a concentration of 2 OD, a much higher number of MSCs accumulated near the magnet (Fig. 2D).

The distribution of fluorescent signal from labeled MSCs around the entire TM circumference was quantified for at least 3 injection experiments for each condition and averaged, as shown in polar histograms (Fig. 2E–H). Kuiper’s V tests for non-uniform cell distribution at the site of the magnet (0°) demonstrated that 20 nm PBNC-MSCs incubated at a PBNC solution concentration of 10 OD and 200 nm PBNC-MSCs incubated at a PBNC solution concentration of 2 OD resulted in significant cell accumulation adjacent to the magnet. While 20 nm PBNC-MSCs at a PBNC solution concentration of 2 OD were steered to the magnet location, the total cell signal at the magnet site was similar to control eyes (Fig. 2I). Only 200 nm PBNC-MSCs at a solution concentration of 2 OD resulted in significantly higher fluorescent cell signal at the magnet site compared to unlabeled MSC delivery (Fig. 2I). We do not entirely understand why 20 nm PBNCs did not produce more efficient cell steering. However, we can likely rule out any impact of 20 nm PBNCs on cell viability as a cause, since PBNC-MSCs were injected only after being washed, detached, and counted, which would have likely eliminated most non-viable (lysed) cells. In any case, we conclude that 200 nm PBNCs are preferred for improving cell delivery to the TM.

### Optimizing magnetic field exposure time

**Figure 3:**
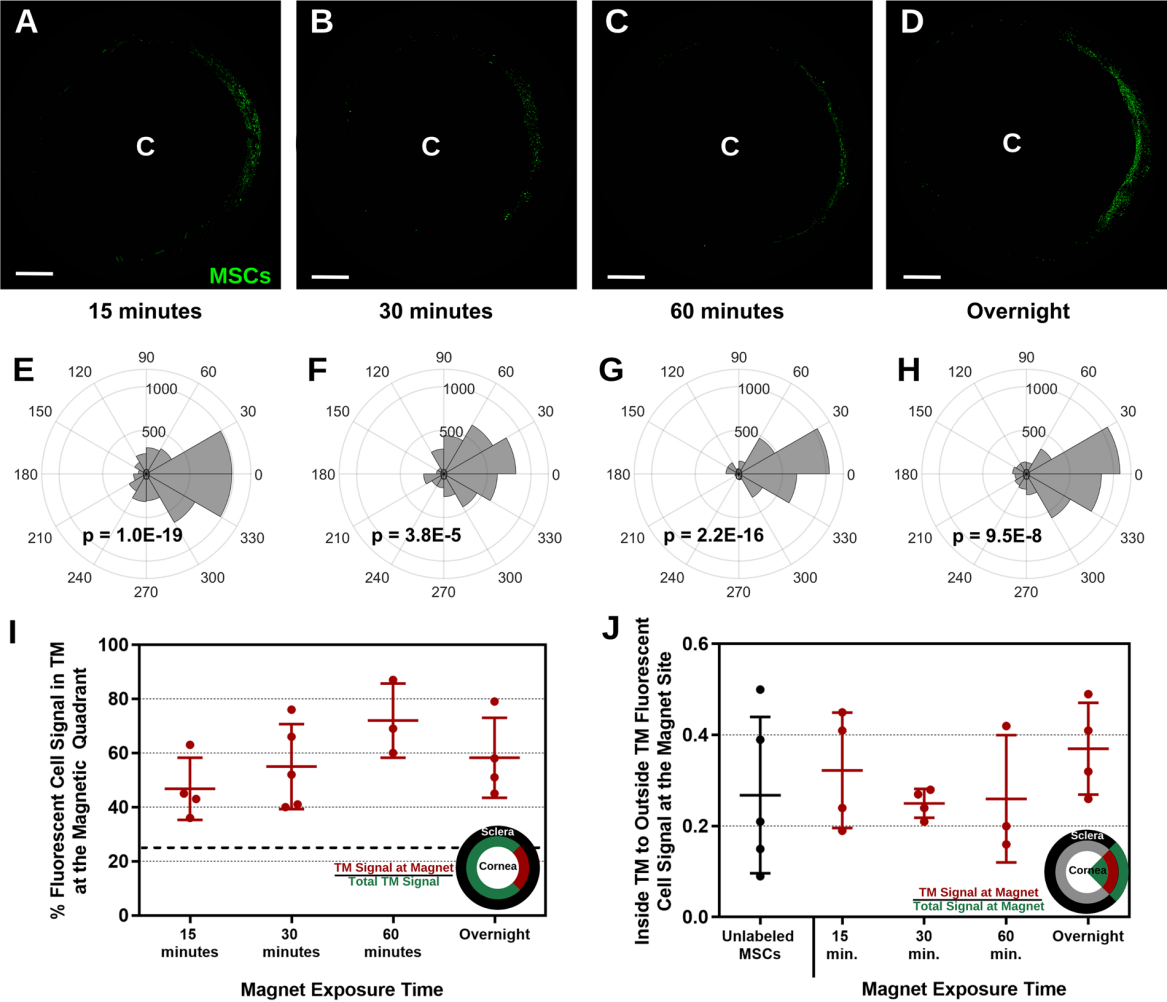
Effect of Magnetic Field Exposure Time on Steering of 200 nm PBNC-MSCs to the TM. **(A–D)** Representative *en face* micrographs of the anterior region of the eye after MSC delivery. Bar magnets were placed near the limbal region overnight (Fig. 2B). “C” denotes the approximate center of the cornea. Note that the image has been masked so that only signal from the putative TM region is shown (see Methods). Scale bars denote 2 mm. **(E–H)** The polar histograms illustrate the total fluorescence intensity within the TM region (plotted on the radial coordinate, in arbitrary units) for 30° sectors around the eye. p-values were calculated using Kuiper’s V test to assess if distribution was non-uniformly skewed towards the center of the magnet location (0°). **(A,E)** 15 minutes (n = 4 eyes), **(B,F)** 30 minutes (n = 5 eyes), **(C,G)** 60 minutes (n = 3 eyes), and **(D,H)** overnight (n = 4 eyes) magnet exposure experiments are shown. **(I)** Percentage of fluorescent cell signal in the TM in the magnet region, defined as the wedge extending from 45° to −45°. Dotted line indicates the value that would be expected if there were uniform cell delivery to the entire TM circumference (25%). **(J)** Fluorescent signal in the TM region relative to the fluorescent signal not in the TM region, both within the magnet site, i.e. within the sector extending from −45° to +45°. Individual data points are shown with central bars indicating mean values and error bars denoting standard deviation. All comparisons between groups were not significant (p > 0.05) as determined by one-way ANOVA with post hoc Tukey analysis.

It was encouraging that an external magnet could steer labeled MSCs to the TM, but overnight magnet exposure would be problematic for future clinical translation. Thus, we assessed how magnet exposure affected cell delivery to the TM, using MSCs labeled with 200 nm PBNCs at a solution concentration of 2 OD. Prior to MSC injection, a bar magnet was placed adjacent to the limbus for either 15 minutes, 30 minutes, 60 minutes, or overnight (representative micrographs, Fig. 3A–D). The cell distributions near the magnet were similar for all durations, each resulting in significant PBNC-MSC accumulation adjacent to the magnet (Fig. 3E–H).

To further quantify cell steering, total fluorescent signal in the TM within a 90° sector (45° on both sides of the magnet center at 0°) was quantified and compared to fluorescent signal in the entire TM (Fig. 3I). We found that 50 to 75% of MSCs in the TM region were steered to the magnet site and that no significant differences were observed between the durations tested. This suggests that PBNC-MSCs are retained in the TM since, after 15, 30, and 60 minute magnet exposure, anterior segments were perfused overnight. Thus, If MSCs were not retained at least overnight in the TM, the results for shorter magnet durations would be much worse than those for overnight magnetic exposure, which was not what was observed.

Further, to assess how many cells were delivered to non-target tissues in the magnet region, we computed a quantity called “TM specificity”, defined as the ratio of fluorescence within the TM to fluorescence outside the TM (i.e. anterior or posterior to the TM margins), both within the 90° sector adjacent to the magnet. TM specificity was similar for all tested durations (Fig. 3J). We conclude that cell steering occurs rapidly, i.e. a 15-minute magnet placement is sufficient to steer many cells to the TM.

### Improving delivery efficiency to the entire TM with PBNCs

**Figure 4:**
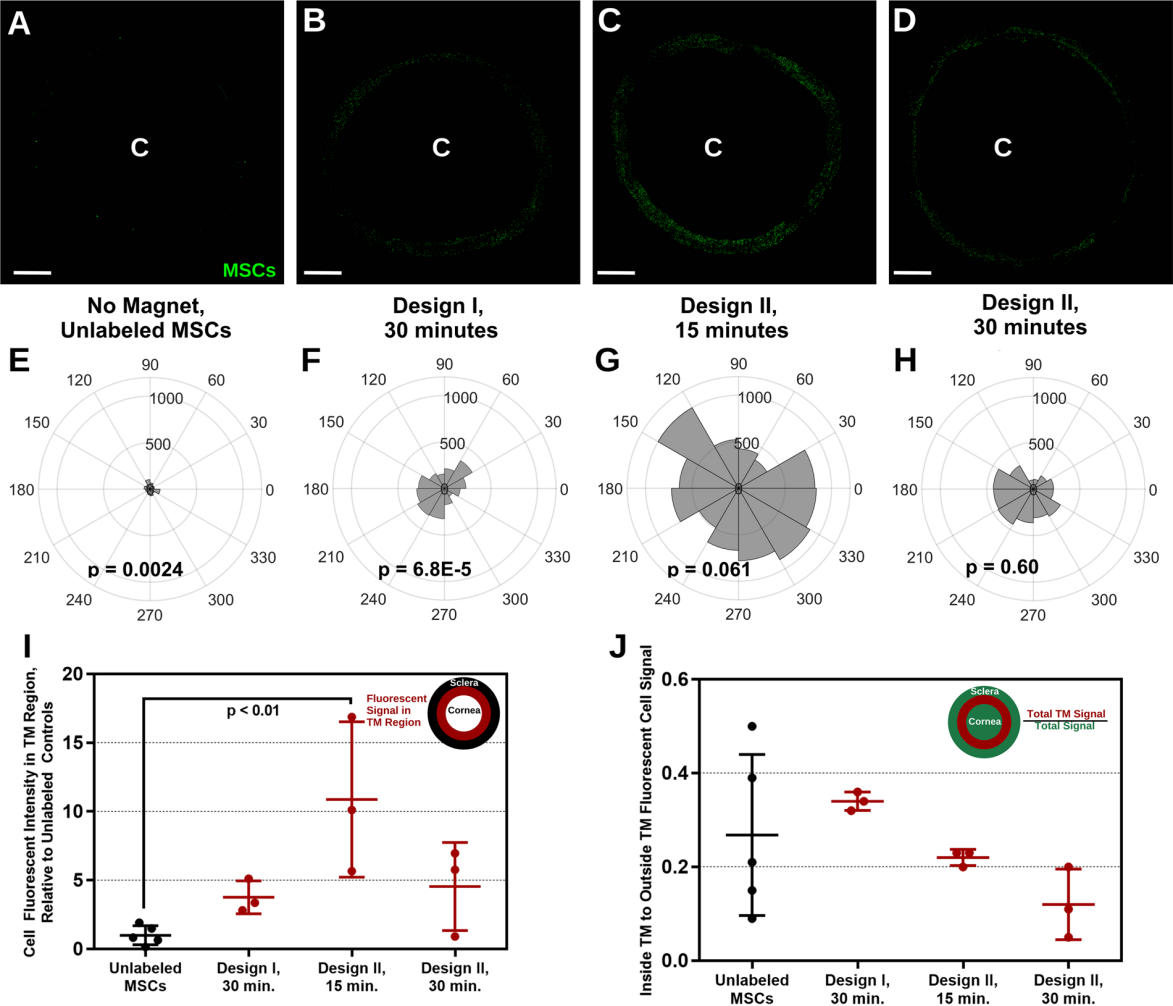
360° Delivery of PBNC-MSCs Using Ring Magnets. **(A–D)** PBNC-MSCs were injected into eyes with two types of ring magnets placed around the circumference for different durations. Representative *en face* micrographs are shown. “C” denotes the approximate center of the cornea. Note that the image has been masked so that only signal from the putative TM region is shown (see Methods). Scale bars denote 2 mm. **(E–H)** The polar histograms illustrate the total fluorescence intensity within the TM region (plotted on the radial coordinate, in arbitrary units) for 30° sectors around the eye. P-values were calculated using Rayleigh’s test to assess uniform circumferential delivery (p < 0.05 denotes non-uniform delivery). **(A,E)** no magnet (n = 5 eyes), **(B,F)** Magnet Design I for 30 minutes (n = 3 eyes), **(C,G)** Magnet Design II for 15 minutes (n = 3 eyes), and **(D**,**H)** Magnet Design II for 30 minutes (n = 3 eyes) experiments are shown. **(I)** Quantification of cell signal in the TM relative to signal from eyes injected with unlabeled MSCs. **(J)** MSC delivery specificity to the TM, i.e. fluorescent signal in the TM region relative to the fluorescent signal outside the TM (i.e. anterior or posterior to the TM margins). Individual data points are shown with central bars indicating mean values and error bars denoting standard deviation. Significant differences (p < 0.05) between groups were determined by one-way ANOVA with post hoc Tukey analysis.

The above results show that PBNC-MSC delivery to a single quadrant of the eye can be improved by use of a magnet. We then asked whether delivery could be improved to the entire circumference of the eye. 250,000 MSCs were injected as described previously and steered using one of two different ring magnet designs: Design I, a 3D printed magnet holder (approximate 10mT field strength at the center of eye), and Design II, an axial polarized ring magnet (approximate 30mT field strength at the center of eye) (Supplementary Fig. 3). Design I was tested for a 30 minute magnet exposure, while Design II was assessed for both 15 and 30 minute exposures (representative micrographs shown in Fig. 4A–D). Each configuration resulted in increased fluorescent signal from MSCs around the TM circumference compared to control injections (Fig. 4E–H). With exposure to a ring magnet we expect uniform delivery to the entire TM circumference, however, only Design II with exposures of 15 and 30 minutes did not result in a statistically significant non-uniform distribution, as determined by Rayleigh’s test for uniform circular distribution. The greatest cell delivery was observed for Design II, 15 minute exposure, which resulted in as much as a 10x increase in cell delivery compared to controls (Fig. 4I). TM specificity was computed as above, except over the entire 360 degree circumference of the eye. Design I had higher TM specificity compared to Design II at both 15 and 30 minute magnetic field exposure (Fig. 4J). We conclude that PBNC-MSCs can be steered to the entire TM circumference in as little as 15 minutes, and further optimization of the magnetic field would be desirable to increase delivery specificity to the TM.

### Histological assessment of the TM After PBNC-MSC delivery

**Figure 5:**
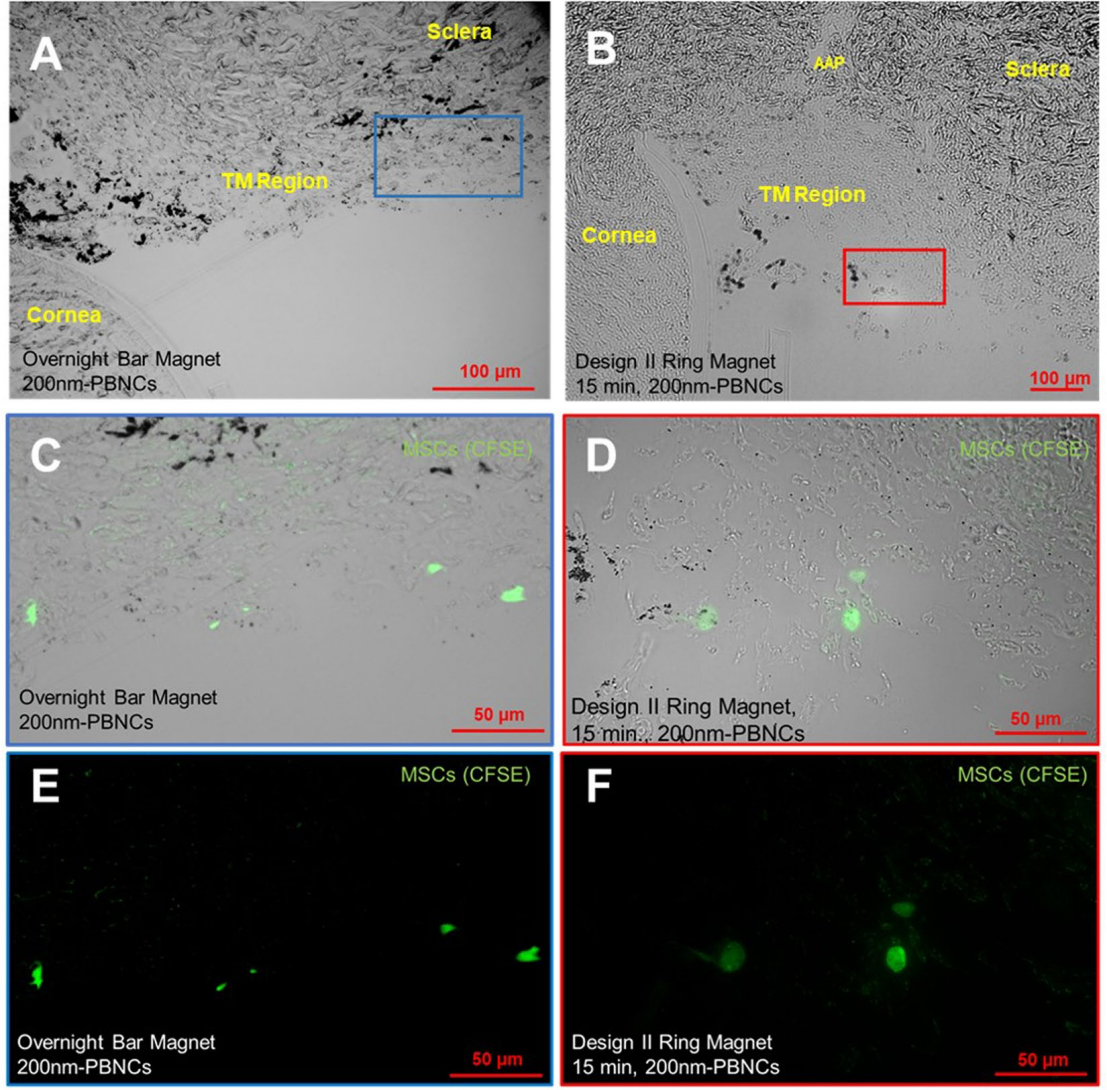
Histological Assessment of PBNC-MSC Delivery to the TM. Representative sagittal sections of the TM region showing fluorescently labeled PBNC-MSCs (green) for **(A,C,E)** overnight bar magnet exposure and **(B,D,F)** Design II, 15 minute ring magnet experiments. **(A,B)** Lower magnification brightfield micrographs are labeled with relevant anatomical features for orientation (AAP = angular aqueous plexus, an analogue of Schlemm’s canal in porcine eyes). The AAP is discontinuous and not present in every section. The TM is identified based on its porous microstructure, relative location with respect to the AAP (when present) and relative location with respect to the limbus (corneal – scleral border). Approximate zoomed regions are shown as blue and red boxes and magnified in panels **(C,D)** and with the brightfield overlay removed **(E,F)**.

*En face* micrographs lack the detail required to properly identify where cells are delivered within the TM. Thus, anterior segments were processed and resin-embedded, and 2 µm thick sagittal sections through the TM were cut. Representative images for overnight bar magnet and 15 minute Design II ring magnet exposures show 200 nm PBNC-MSCs in the TM, identifiable by the porous tissue structure and by anatomical landmarks (Fig. 5A–F). Most of the cells remained on the inner TM structures as opposed to penetrating deeper into the tissue. We conclude that PBNC-MSCs are indeed delivered to the TM.

### Toxicity of PBNCs

**Figure 6:**
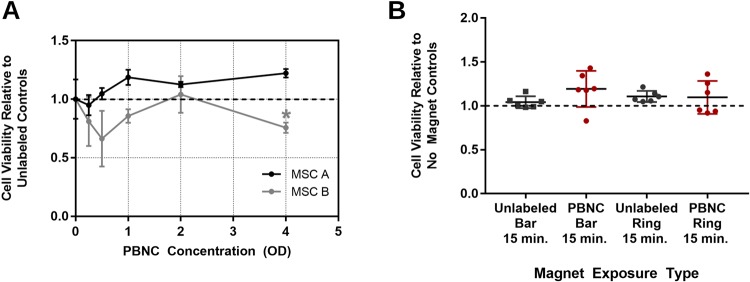
Cell Viability Results for MSCs labeled with 200 nm PBNCs. **(A)** Relative viability 1 day after 200 nm PBNC labeling for 2 MSC donor strains as determined by flow cytometry (n = 3 technical replicates for each OD). **(B)** Relative viability of MSCs after magnet exposure for 15 minutes. Bar and Design II ring magnets were placed adjacent to unlabeled and 200 nm PBNC-labeled cells in suspension. Individual data points for each group are shown with horizontal lines indicating means (n = 6 technical replicates for each condition). Error bars denote standard deviation. Asterisks denote significant difference (p < 0.05) compared to unlabeled controls as determined by ANOVA, with Dunnett’s post hoc analysis.

We were concerned that PBNCs might adversely affect MSC viability. Thus, MSCs were incubated with different concentrations of 200 nm PBNC solutions overnight. Cellular viability 1 day post-PBNC incubation was assessed by propidium iodide staining and flow cytometry to quantify any cytotoxic effects of incubating MSCs with PBNCs. Minimal PBNC toxicity was observed after 1 day for both MSC donor strains across the tested PBNC concentrations (Fig. 6A). We also assessed MSC viability after magnetic field exposure, with and without PBNC labeling. Unlabeled and 200 nm PBNC-labeled MSCs were suspended in cell culture media at 50,000 cells/mL in 20 mL syringes and exposed to a bar or Design II ring magnet for 0 or 15 minutes to approximately mimic how cells experienced magnetic forces during experiments in anterior segments. Exposure of unlabeled and PBNC-labeled MSCs to either magnetic field did not result in reduced viability compared to controls without magnetic exposure (Fig. 6B). We conclude that neither the PBNCs nor the 15 minute magnet exposure were toxic to the MSCs.

### Effect of PBNCs on MSC multipotency

**Figure 7:**
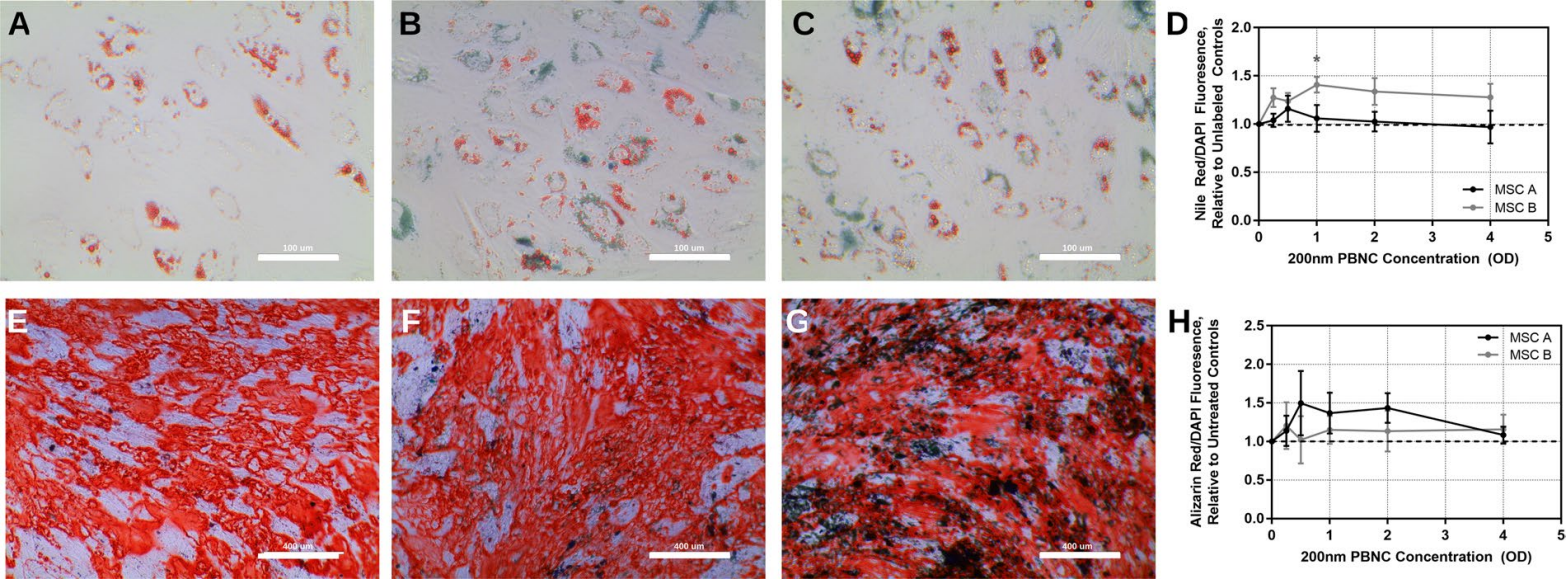
Effect of 200 nm PBNCs on MSC Differentiation. **(A–D)** Oil Red O staining for lipids 7 days after adipogenesis treatment in **(A)** unlabeled MSCs, **(B)** 1 OD 200 nm PBNC-MSCs, and **(C)** 4 OD 200 nm PBNC-MSCs. **(D)** Quantification of ratio of fluorescent Nile Red (lipids) to DAPI (nuclei) signal relative to untreated controls for 2 stem cell lines (n = 3 technical replicates for each concentration). **(E–H)** Alizarin Red O staining for free calcium after 21 day osteogenesis treatment in **(E)** unlabeled MSCs, **(F)** 1 OD 200 nm PBNC-MSCs, and **(G)** 4 OD 200 nm PBNC-MSCs. **(H)** Ratios of fluorescent Alizarin Red (free calcium) to DAPI (nuclei) signal relative to untreated controls for 2 stem cells (n = 3 technical replicates for each concentration). Error bars denote standard deviations. Asterisks denote significant difference (p < 0.05) compared to unlabeled controls as determined by ANOVA, with Dunnett’s post hoc analysis.

Finally, we asked whether the presence of PBNCs might adversely affect multipotency of MSCs. MSCs were treated with adipogenic medium for 7 days after PBNC labeling and qualitatively assessed for differentiation to adipocytes by Oil Red O staining (Fig. 7A–C). Positive lipid staining was found for all PBNC concentrations for 2 MSC donor cell lines. Further, cells were stained with Nile Red fluorescent stain and DAPI nuclei stain. Nile Red signal normalized to DAPI intensity resulted in similar ratios for each donor strain and PBNC concentration, indicating minimal effect of PBNCs on adipogenesis (Fig. 7D).

MSCs were also differentiated towards an osteogenic phenotype for 21 days, followed by staining with Alizarin Red to detect free calcium (Fig. 7E–G). Alizarin Red staining was detected consistently in all experimental conditions. Alizarin Red fluorescent intensity normalized to DAPI fluorescent intensity was similar for all PBNC concentrations and for both MSC donor strains, indicating minimal effect of PBNCs on osteogenesis (Fig. 7H).

## Discussion

Stem cells have the potential to refunctionalize the damaged TM in glaucoma patients and thus restore IOP homeostasis. To achieve this goal, it is highly desirable to optimize cell delivery to the TM. Previous studies in which cells were injected into the anterior chamber and then passively carried to the TM by aqueous humor flow showed that stem cells were undetectable in the TM after only 4 days, or showed significant stem cell delivery to unwanted sites, such as the cornea and iris^16,17^. In addition, segmental flow to the TM makes delivery to the entire TM circumference impossible if cells are driven by passive aqueous humor outflow, a problem that is exacerbated in glaucoma patients. We thus investigated active stem cell delivery to the TM. Specifically, we assessed whether magnetic nanoparticles could be utilized to steer MSCs to the TM in the presence of a magnetic field.

We tested two PBNC sizes, since particle size was hypothesized to impact cell steering. We observed that both PBNC sizes were capable of labeling MSCs, but that 200 nm PBNCs were more effective at steering MSCs to the TM than 20 nm PBNCs. This is consistent with the fact that larger magnetic particles are expected to be more responsive to a magnetic field.

Regenerative medicine therapies rely on MSCs to restore tissue function through paracrine signaling or cellular differentiation, so it is important that PBNCs not adversely affect MSC function if they are to be used in practice. Fortunately, 200 nm PBNCs did not reduce MSC viability, nor did they adversely affect osteogenesis and adipogenesis. One goal of MSC therapies for the TM is for MSCs to differentiate into TM cells after delivery. While differentiation of labelled MSCs to a TM-like phenotype has not been established here, evidence that PBNCs do not alter traditional differentiation pathways suggests that MSC to TM cell differentiation will also not be affected.

For *in vivo* usage and eventual clinical translation, it is advantageous to have a magnet exposure time that is as short as possible. We observed that 200 nm PBNC-MSCs could be steered to the TM in as little as 15 minutes, with additional exposure time resulting in little net benefit. Further, since the shortest exposure tested was still effective, it is possible that even shorter durations might be effective.

Excitingly, MSCs labeled with 200 nm PBNCs could be magnetically steered to the entire TM circumference. We observed some degree of uniform cell delivery for both ring magnet configurations tested, with Design II at 15 and 30 minute exposures resulting in more cell signal in the TM compared to Design I at 30 minutes. However, Design I had higher TM specificity compared to Design II. The differences between these two ring-magnet designs can help inform magnetic field design for future work. Design II was a commercially-available, axially-polarized ring magnet, meaning most of the pulling force was in the axial (i.e. anterior-posterior) direction; such a field is not optimal for steering MSCs to the TM, which would instead require forces on injected cells to be primarily in the radial direction. Indeed, a radially-directed magnetic force was present only near the top and bottom faces of the magnet, and, while one of the faces was near the limbus, the other was not since the magnet thickness was much greater than the TM height, i.e. the anterior-posterior extent of the TM. As a result, this likely led to undesired cell delivery near the second magnet face.

However, one advantage of the Design II ring magnet was that the magnet was in direct contact with the limbus, presumably creating high field strengths near the TM. This was unlike Design I, in which 10 smaller magnets were positioned in a 3D-printed holder. Although the overall magnetic pulling force was directed towards the TM in Design I, the plastic holder resulted in a gap between the magnet and eye surface, reducing the magnetic field strength (10mT [Design I] and 30mT [Design II] field strength at the center of the eye as measured by a Gaussmeter). This in turn presumably reduced cell delivery efficiency. Conversely, with the overall magnetic pull force focused at the TM region in Design I, higher TM specificity was obtained. This discussion highlights the potential benefits of optimizing the magnetic field, which presumably will be useful in delivering labelled cells efficiently to the entire TM with minimal delivery to unwanted tissues.

This work is subject to certain limitations. First, while MSC delivery to the TM was significantly improved, the ultimate goal is differentiation of MSCs to functional TM cells, which we have not shown, or delivery of pre-differentiated TM cells. Since the PBNCs did not impact cell viability and tested differentiation pathways, we do not anticipate TM differentiation being impacted, but this remains to be shown. Second, the ring magnet design was far from optimal, and for testing this approach in small animal models and eventual clinical translation, the magnetic field and PBNC labeling should be further optimized. Specifically, by using magnets with a field more focused into the TM and possibly by altering the PBNC concentration used for MSC labeling, more precise delivery of MSCs to the TM region with minimal unwanted delivery to other ocular tissues should be possible. It would also be of interest to “pre-perfuse” eyes with a tracer^32,33^ to confirm that magnetically-guided cell delivery is relatively independent of segmental flow patterns through the TM. We expect that, in view of the marked magnetic steering impact of 200 nm PBNCs, segmental flow will not markedly affect steering, but direct experimental validation would be useful. In future work, we plan to use combined ultrasound-photoacoustic imaging of the PBNC-labeled MSCs to track delivery and engraftment of MSCs into the TM region, simplifying optimization of this technology in animal models of glaucoma for eventual therapeutic experiments.

In conclusion, PBNCs were found to be suitable for MSC labeling and for helping steer cells uniformly to the TM in as little as 15 minutes, unlike the situation with passive delivery. This labeling technology has the potential to improve regenerative medicine therapies for the TM in glaucoma patients.

## Methods and Materials

### Mesenchymal stem cell sourcing

Human adipose derived mesenchymal stem cells (MSCs, Lonza, Basel, Switzerland were maintained in α-minimum essential medium containing 20% fetal bovine serum (FBS), penicillin, streptomycin, and L-glutamine. At 80–90% confluency, MSCs were detached with 0.05% Trypsin-EDTA (w/v, Cellgro, Corning, Corning, NY, USA) and seeded onto fresh cell-culture T75 flasks at 5,000 cells/cm^2^. MSCs were used at passage numbers five or six for all experiments. MSC donor strains (MSC A, 51 years/female, and MSC B, 44 years/female) were validated by Lonza for expression of MSC surface markers (CD13, CD29, CD44, CD73, CD90, CD105, and CD166) and minimal expression of negative MSC markers (CD14, CD131, CD45). MSCs were also previously characterized for adipogenic, chondrogenic, and osteogenic differentiation and CD90 expression following expansion^34^.

### PBNC nanoparticle synthesis and characterization

PBNCs were synthesized according to methods described previously^26^. 150 mL of 0.04% (w/v) dextran-coated iron oxide (Fe_3_O_4_) nanoparticles in deionized ultra-filtered water were vigorously mixed with 5 mL of 5% w/v potassium hexacyanoferrate(II) trihydrate (K_4_Fe(CN)_6_·3H_2_O, Sigma-Aldrich, St. Louis, MO, USA). After one minute, 2.496 mL of 1.85% (v/v) hydrochloric acid (HCl, Sigma-Aldrich, St. Louis, MO, USA) was added. To alter PBNC size, the diameter of the iron oxide nanoparticles was altered. Dextran-coated iron oxide nanoparticles with a diameter of 10 nm (Ocean NanoTech, San Diego, CA, USA) produced 200 nm PBNCs. Iron oxide nanoparticles with a diameter of 2 nm produced 20 nm PBNCs. Transmission electron microscopy (TEM) (Hitachi HT7700 TEM, IEN/IMAT Materials Characterization Facility, Georgia Institute of Technology) was used to visualize particle size and shape (Supplementary Fig. 1). PBNCs were sterilized under UV light for at least 12 hours prior to all experiments.

### MSC fluorescent and PBNC labeling

MSCs at 80–90% confluence were incubated with PBNCs in cell culture media at PBNC concentrations ranging from 0.25 to 10 OD for 24 hours. Unless otherwise noted, 2 OD PBNC concentrations were used in experiments reported herein. Uptake of PBNCs by MSCs was visually detected within cells by light microscopy (EVOS XL, Thermo-Fisher, Waltham, MA, USA). Prior to imaging, PBNC-labeled MSCs were fixed with 10% buffered formalin for 15 minutes followed by cytoplasmic staining with 1% eosin (w/v) for 5 minutes. Triplicate experiments were performed for each PBNC incubating concentration to confirm PBNC uptake, which we defined as PBNC colocalization with eosin-stained cytoplasm. Further characterization of PBNC-MSCs was performed by flow cytometry to assess changes in light scatter known to correlate with nanoparticle uptake^35^. Specifically, labeled MSCs were detached from culture dishes with 0.05% trypsin-EDTA (w/v) and side scatter (SSC) properties were analyzed using an Attune NxT (Thermo-Fisher, Waltham, MA, USA) flow cytometer at 200 µL/min flow rates.

A Vevo 2100/LAZR imaging system, which incorporates ultrasound and photoacoustic (US/PA) imaging modalities (VisualSonics, Inc., Toronto, ON, Canada), was used to verify that PBNC-MSCs could produce a photoacoustic signal. PBNC-MSCs were imaged in a tissue-mimicking gelatin phantom^36^. The base of the phantom consisted of 8% (w/v) gelatin and 0.2% (w/v) silica (Sigma-Aldrich, St. Louis, MO, USA). Gelatin phantom inclusions were made by adding 16% (w/v) gelatin to an equal volume of PBNC-MSCs. Each inclusion was imaged at 5 nm wavelength increments from λ = 680 nm to 970 nm with the US/PA transducer oriented perpendicular to the gelatin base.

For MSC injection experiments, MSCs were detached from culture flasks with 0.05% trypsin-EDTA (w/v), centrifuged, and resuspended in 5 µM carboxyfluorescein succinimidyl ester (CFSE, affymetrix eBioscience, Santa Clara, CA, USA) in PBS after PBNC uptake (for PBNC-MSC samples). After incubating for 15 minutes at 37 °C, media was added to quench any unreacted CFSE before injection of MSCs into the eye.

### Organ cultured porcine eyes

Anterior segment organ culture is a well-established method to maintain trabecular meshwork function *ex vivo* for several weeks, and we adopted this approach for our work here^30,31,37–39^. Fresh porcine eyes from a slaughterhouse (Holifield Farms, Covington, GA, USA) were dissected within 6–8 hours of enucleation to isolate the outflow tissues and anterior corneoscleral shell, similar to previously reported methods^37,38^. Briefly, orbital connective tissue was dissected away from the eye globe, and eyes were soaked in Betadine (Purdue Pharma, New York City, NY, USA) solution for 5 minutes, after which eyes were transferred to a sterile laminar flow hood for the remainder of the process. Eyes were washed with sterile PBS and hemisected with a razor blade to isolate the anterior half of the eye. The vitreous humor and lens were removed. Next, the iris was cut radially back to the iris root and pectinate ligaments until the TM was revealed. The ciliary processes were then carefully removed while preserving the TM. Any remaining vascularized or pigmented tissue was removed from scleral and cornea surfaces with sterile cotton swabs.

Dissected anterior segments were placed in custom-built organ culture dishes and clamped in place (Supplementary Fig. 2). Organ culture dishes were placed in a sterile 37 °C humidified incubator and perfused at 2.5 µL/min with serum-free media containing penicillin, streptomycin, and amphotericin. A calibrated pressure transducer (142PC01G, Honeywell, Morris Plains, NJ, USA) monitored pressure within the anterior segment and readings were recorded every 60 seconds in LabVIEW software (2014 sp1, National Instruments, Austin, TX, USA). Eyes were stabilized for at least 48 hours before conducting any experiments. Any eye which did not demonstrate a stable outflow facility (the ratio of perfusion flow rate over IOP, and a measure of TM function) between 0.45 and 0.125 µL/min/mmHg (pressures of ~6 mmHg to 20 mmHg IOP) were considered as outliers and not used for MSC delivery experiments (Supplementary Fig. 2).

### PBNC-MSC magnetically steered delivery

PBNC-MSCs were detached from culture flasks using 0.05% trypsin-EDTA (w/v) and counted to determine cell number (Attune NxT flow cytometer, Thermo Fisher, Waltham, MA, USA). After fluorescent staining with CFSE, cells were resuspended in serum-free organ culture media at 1 million cells/mL. 250 µL of cell suspension was injected with a 31-gauge needle through the cornea (5–10 second injection duration) while anterior segments were pressure clamped at 8–12 mmHg. We chose to inject 250,000 cells for several reasons. First, a previous study used 500,000 cells^40^. We tested 250,000, 500,000 and 1,000,000 cells in preliminary studies, but found that larger cell numbers did not lead to more signal in the TM, possibly due to saturation of cell adhesion sites in the TM (data not shown). Immediately after injection, anterior segments were again perfused at 2.5 µL/min. For magnetic delivery experiments, cells were steered in one of two ways.

1. For initial optimization experiments, neodymium rare earth bar magnets (0.75″ length × 0.25″ width × 0.25″ height, 40 mT field strength near center of eye, K&J Magnetics, Pipersville, PA, USA) were placed next to one quadrant of the anterior segments near the limbal (corneal-scleral border) region prior to MSC injection. The magnetic field strength near the center of the eyes was approximately 40 mT, as determined by a Gaussmeter (Lake Shore Cryotronics, Inc., Westerville, OH, USA).
2. For 360° delivery experiments, two different ring magnets were used (Supplementary Fig. 3). First, a 3D-printed magnet holder containing 10 axially-polarized cylindrical magnets (3/16″ diameter × 0.25″ height, 10 mT field strength near center of eye, K&J Magnetics, Pipersville, PA, USA) was created (referred to as Design I). Alternatively, a commercially available axially polarized ring magnet (1.5″ outer diameter × 0.75″ inner diameter × 0.25″ height, 30 mT field strength near center of eye, K&J Magnetics, Pipersville, PA, USA) was used (referred to as Design II). Ring magnets were placed around the limbus before MSC injections and removed at specified time points.

Following MSC injection and magnet exposure for the indicated time, anterior segments were perfused overnight, after which they were removed from organ culture and immersed in 10% buffered formalin overnight (4 °C) to fix tissue.

### Fluorescence microscopy and quantification

After fixation, the TM region of anterior segments was fluorescently imaged *en face*. Anterior segments were placed in PBS on a 50 mm glass-bottomed dish, after scleral tissue posterior to the TM was removed so that the TM was in contact with the dish surface to improve imaging. Micrographs of the entire TM circumference were captured by confocal microscopy (LSM 700, Carl-Zeiss, Oberkochen, Germany) to identify CFSE-tagged MSCs delivered to the limbal region. Brightfield overlays were also captured to identify the corneal margin and hence the approximate location of the TM. Micrographs were captured at 50x magnification as z-stack tile scans to account for TM depth and height differences around the circumference of the anterior segment. Maximum intensity projections were created for each *en face* image for use in further image quantification.

To specify the TM region within which quantification was carried out, masks were created from brightfield images using the corneal margin as a landmark (Supplementary Fig. 4). A 1 mm ring from the edge of the cornea was defined, using ImageJ^41,42^, to be the TM region within each image. This size is approximately twice the reported anterior-posterior dimension of the TM^43^; we chose a somewhat larger region since we could not determine the exact anterior boundary location of the TM and wanted to ensure that our analysis region contained the TM. Images were further processed in MATLAB (MathWorks, Natick, MA, USA) to remove fluorescent debris appreciably larger than a typical cell size (size greater than 100 µm). To quantify fluorescent intensity around the TM circumference, a polar coordinate system was overlaid onto images and the total CFSE intensity was quantified within each one degree sector. Intensities were then summed over 30° sectors, resulting in 12 intensity values for each image. The process was then repeated, considering only the signal outside the masked region, to determine the cell signal not located in the TM. For print publication purposes, the brightness was increased for all representative micrographs in a consistent manner using Photoshop (Adobe Systems, San Jose, CA, USA).

### Histology

Anterior quadrants of fixed porcine eyes were cut into 3 mm wide meridional wedges and trimmed to isolate the outflow region. The tissue was dehydrated through an ethanol series, infiltrated and embedded in Histocryl media (Electron Microscopy Sciences, Hatfield, PA, USA). Two micron thick sections were cut on a UC7 ultramicrotome (Leica, Wetzlar, Germany) with a glass knife and examined with a DM6 Epifluorescent microscope (Leica). Sections were examined to visualize CFSE-labeled stem cells, using the green fluorescent protein filter cube.

### Live-dead assessment of effects of pbncs on mscs *in vitro*

To assess potential acute toxicity due to PBNCs, MSCs were incubated with PBNCs at various concentrations for 24 hours. Media was removed and propidium iodide (Thermo Fisher, Waltham, MA, USA) was added to each sample for 5 minutes to stain dead, adherent cells. Propidium iodide staining solution was removed and 0.05% trypsin-EDTA (w/v) was added for 5 minutes to detach cells. Media containing 20% FBS was added to neutralize trypsin activity and cells were collected in deep-well 96-well plates (Corning, Corning, New York, USA). Cell samples were processed by flow cytometry by collecting equal volumes from each sample (Attune NxT flow cytometer, Thermo Fisher, Waltham, MA, USA). PBNC-labeled living cell counts (propidium iodide negative), were compared to unlabeled, control living cell counts to determine PBNC toxicity 1 day after PBNC incubation. This approach bypassed the issue of tracking detached, dead cells *in vitro* and determined only how many fewer living cells were present in samples relative to unlabeled controls.

Finally, we assessed whether magnetic fields might interact with PBNCs to lead to cell toxicity. MSCs were either unlabeled, or pre-labeled with 200 nm PBNCs at a concentration of 2 OD. These cells were then suspended at 50,000 cells/mL, and 2 mL of this suspension was loaded into 20 mL syringe barrels having a diameter similar to that of organ-cultured anterior segments, approximately mimicking the situation in cell delivery experiments. Syringes were exposed to a single bar magnet or Design II ring magnet for 15 minutes. After magnet exposure, 2 mL of 2x Propidium Iodide stain was added to each syringe barrel. After five-minute incubation, cell suspensions were transferred to 96 well plates for flow cytometric analysis. Propidium iodide-negative PBNC-labeled and unlabeled MSC counts for each magnet duration were compared to counts of control cells that were not exposed to magnetic fields.

### Stem Cell Multipotency Assessment *in vitro*

We also carried out studies to ensure that PBNC labeling did not negatively affect the multipotency of MSCs. After loading with PBNCs, cells were exposed to adipogenic differentiation or osteogenic differentiation media for 1 or 3 weeks respectively, feeding every three days (Thermo Fisher, Waltham, MA, USA). At their respective time points, cells were fixed in 10% buffered-formalin for 15 minutes. Adipogenesis was quantitatively assessed by Nile Red staining (Thermo Fisher, Waltham, MA, USA)^44^. Cells were incubated for 5 minutes with 100 ng/mL Nile Red (Sigma-Aldrich, St. Louis, MO, USA) in PBS, washed, and probed with DAPI (Thermo Fisher, Waltham, MA, USA). For each cell sample, 16 fluorescent micrographs were captured in a 4 × 4 array across each sample using an imaging plate reader (10x objective, BioTek Cytation 3, Winooski, VT, USA). Nile Red average fluorescent intensities were quantified for each image and normalized to DAPI cell counts. Further, adipogenesis was qualitatively assessed by Oil Red O (Sigma-Aldrich, St. Louis, MO, USA) staining and light microscopy^34^.

To assay for osteogenesis, cells were stained with 2% (w/v) Alizarin Red for 2 minutes followed by PBS rinses to remove excess dye. Experiments were qualitatively assessed by light microscopy, followed by counterstaining cell nuclei with DAPI, and 16 fluorescent micrographs were captured in a 4 × 4 array for each sample using an imaging plate reader as described above.

### Statistics

For *in vitro* experiments, at least three technical replicates were run for each set of experimental conditions. For cell delivery experiments, at least triplicate ocular injections were carried out for every experimental condition using a single MSC donor strain (MSC A or MSC B). Unless otherwise noted, all statistical analyses were performed using GraphPad Prism version 7.04 (GraphPad Software, La Jolla, CA, USA). Analysis of variance (ANOVA) was used to determine if unlabeled, control groups were significantly different from PBNC-labeled groups when applicable. ANOVA makes three assumptions: continuous variables, uniform distribution, and equal variance between groups. For this study, all experimental variables were continuous. Normal distribution was assessed using the Shapiro-Wilk test which tested the null hypothesis that a sample came from a normally distributed population; the null hypothesis was rejected if p < 0.05. The Brown-Forsythe test was used for assessing equal variances where p < 0.05 indicated variances were significantly different.

When ANOVA was suitable, Tukey’s post hoc test was used when comparing all groups or Dunnett’s post hoc test was used when comparing each group mean to a single control mean as indicated. When ANOVA was not suitable, the Kruskal-Wallis test was used with Dunn’s post hoc test. This was required for only one data set (Fig. 2I). Kuiper’s V test was performed using MATLAB to test for non-uniform circumferential cellular distributions in experiments in which cells were steered by a rectangular magnet^45,46^. Average cell signal distribution results for each group were assessed, with p < 0.05 indicating non-uniform skewing of cell distributions toward the magnet location. For experiments in which cells were steered using a ring magnet, Rayleigh’s test for uniform circular distribution was performed using MATLAB for testing cell signal distributions, with p < 0.05 indicating non-uniform cellular distribution around the TM^46^.

## Electronic supplementary material

Supplementary Information

## Data Availability

The datasets generated during and/or analyzed during the current study are available from the corresponding author on reasonable request.
