# Author Correction: Improving Stem Cell Delivery to the Trabecular Meshwork Using Magnetic Nanoparticles

**DOI:** 10.1038/s41598-020-60511-7

**Published:** 2020-02-20

**Authors:** E. J. Snider, K. P. Kubelick, K. Tweed, R. K. Kim, Y. Li, K. Gao, A. T. Read, S. Emelianov, C. R. Ethier

**Affiliations:** 1 Wallace H. Coulter Department of Biomedical Engineering, Georgia Institute of Technology and Emory University, Atlanta, Georgia; 2 School of Electrical and Computer Engineering, Georgia Institute of Technology, Atlanta, Georgia

Correction to: *Scientific Reports* 10.1038/s41598-018-30834-7, published online 16 August 2018

This Article contains errors in the Reference list. Reference 26 is incorrectly listed as ‘Cook, J. R. D., Diego, S., Kubelick, Kelsey, P., Luci, Jeffrey, Emelianov, Stanislav, Y. in *SPIE BiOS*.’ The correct reference 26 appears below as ref. 1.

Additionally, reference 27 is incorrectly listed as ‘Kubelick, K. P., Snider, E. J., Ethier, C. R. & Emelianov, S. Photoacoustic Properties of the Anterior Eye. *Journal of Biomedical Optics* (In Preparation) (2017).’ The correct reference 27 appears below as ref. 2.
