# Supplementary material for: Improving Stem Cell Delivery to the Trabecular Meshwork Using Magnetic Nanoparticles
Source: Sci Rep. 2018 Aug 16;8:12251. doi: 10.1038/s41598-018-30834-7 (PMC6095892; doi:10.1038/s41598-018-30834-7)
Supplement: Supplementary file 1 — Supplementary Information [file 41598_2018_30834_MOESM1_ESM.docx]

# Improving Stem Cell Delivery to the Trabecular Meshwork Using Magnetic Nanoparticles

E.J. Snider^1^, K.P. Kubelick^1^, K. Tweed^1^, R.K. Kim^1^, Y. Li^1^, K. Gao^1^, A.T. Read^1^, S. Emelianov^1,2^, C.R. Ethier^1^*

^1^ Wallace H. Coulter Department of Biomedical Engineering, Georgia Institute of Technology and Emory University, Atlanta, Georgia
^2^ School of Electrical and Computer Engineering, Georgia Institute of Technology and Emory University, Atlanta, Georgia

**Corresponding author**

C. Ross Ethier, Ph.D.

315 Ferst Drive, 2306 IBB, Atlanta, GA 30332-0363

Phone: (404) 385-0100

Fax: (404) 385-1397

Email: [ross.ethier@bme.gatech.edu](mailto:ross.ethier@bme.gatech.edu)

**Supplementary Information**


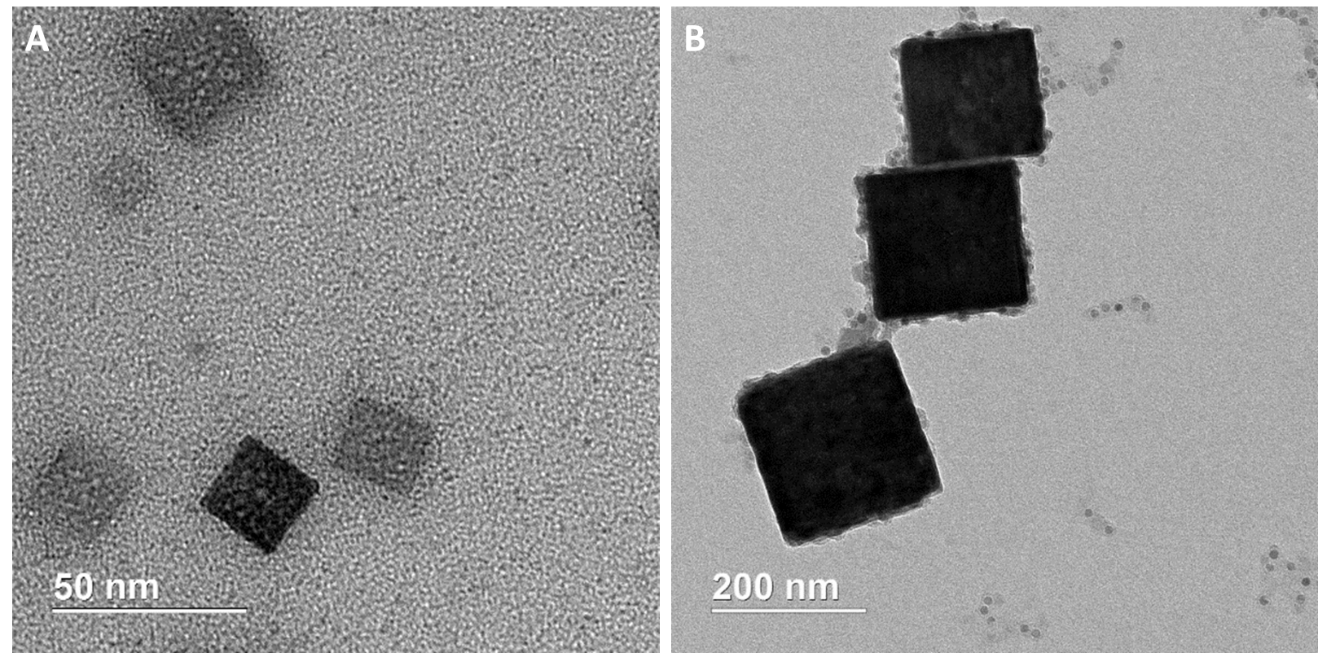


**Supplementary Figure 1. TEM Characterization of PBNCs.** Representative transmission electron micrographs for **(A)** 20nm PBNCs and **(B)** 200nm PBNCs to determine approximate shape and size.


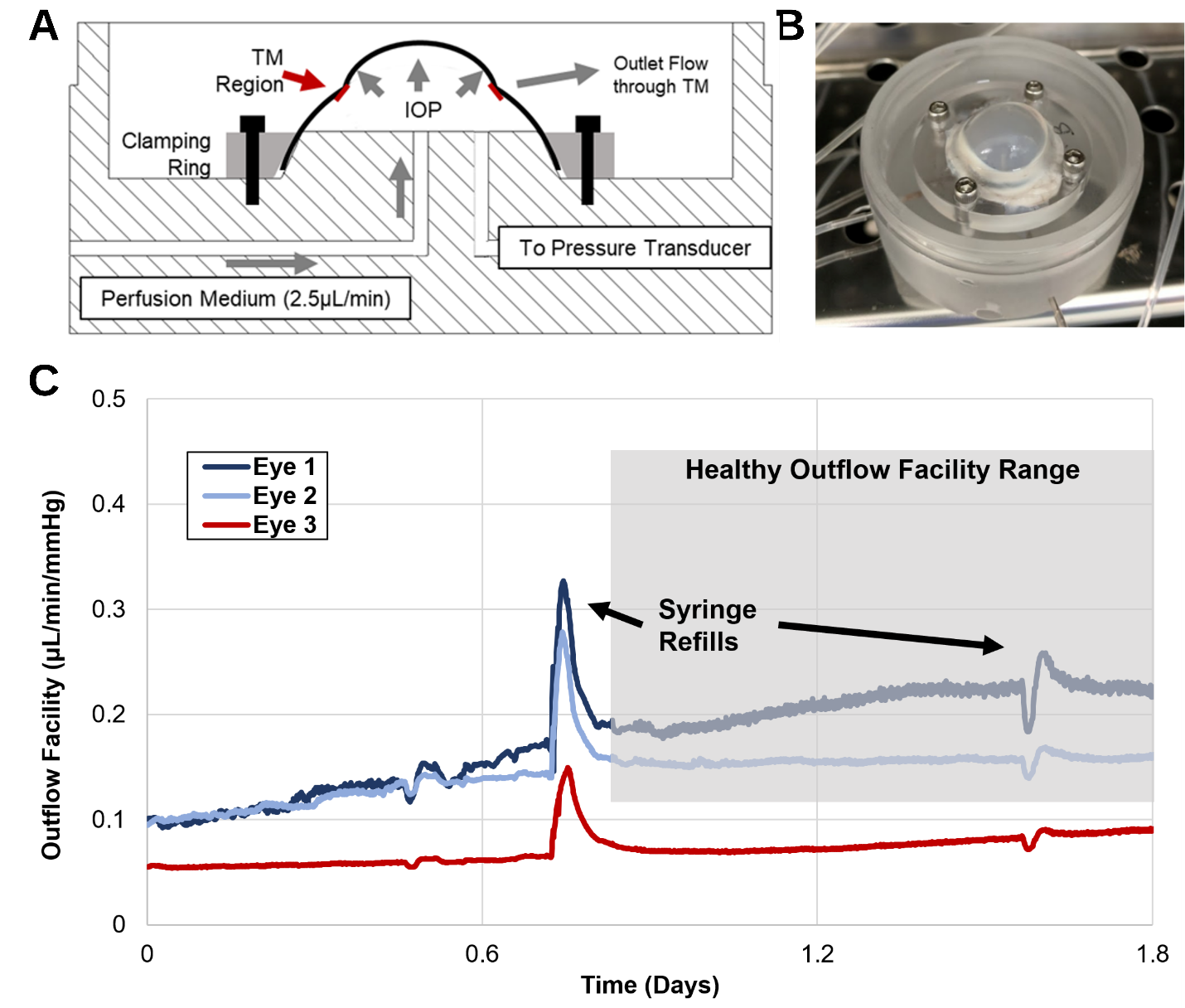


**Supplementary Figure 2. Anterior Segment Organ Culture Setup. (A)** Diagram of porcine anterior segment clamped in organ culture dish. Red label indicates TM region. **(B)** Porcine anterior segment in organ culture after 10 days of perfusion. **(C)** Representative outflow facility traces for three porcine eyes, defined as flow rate (2.5 μl/min) divided by measured IOP. Outflow facility stabilizes after 1-2 days, after which any eyes with outflow facilities outside of the acceptable range were not considered for injection experiments (such as Eye 3).


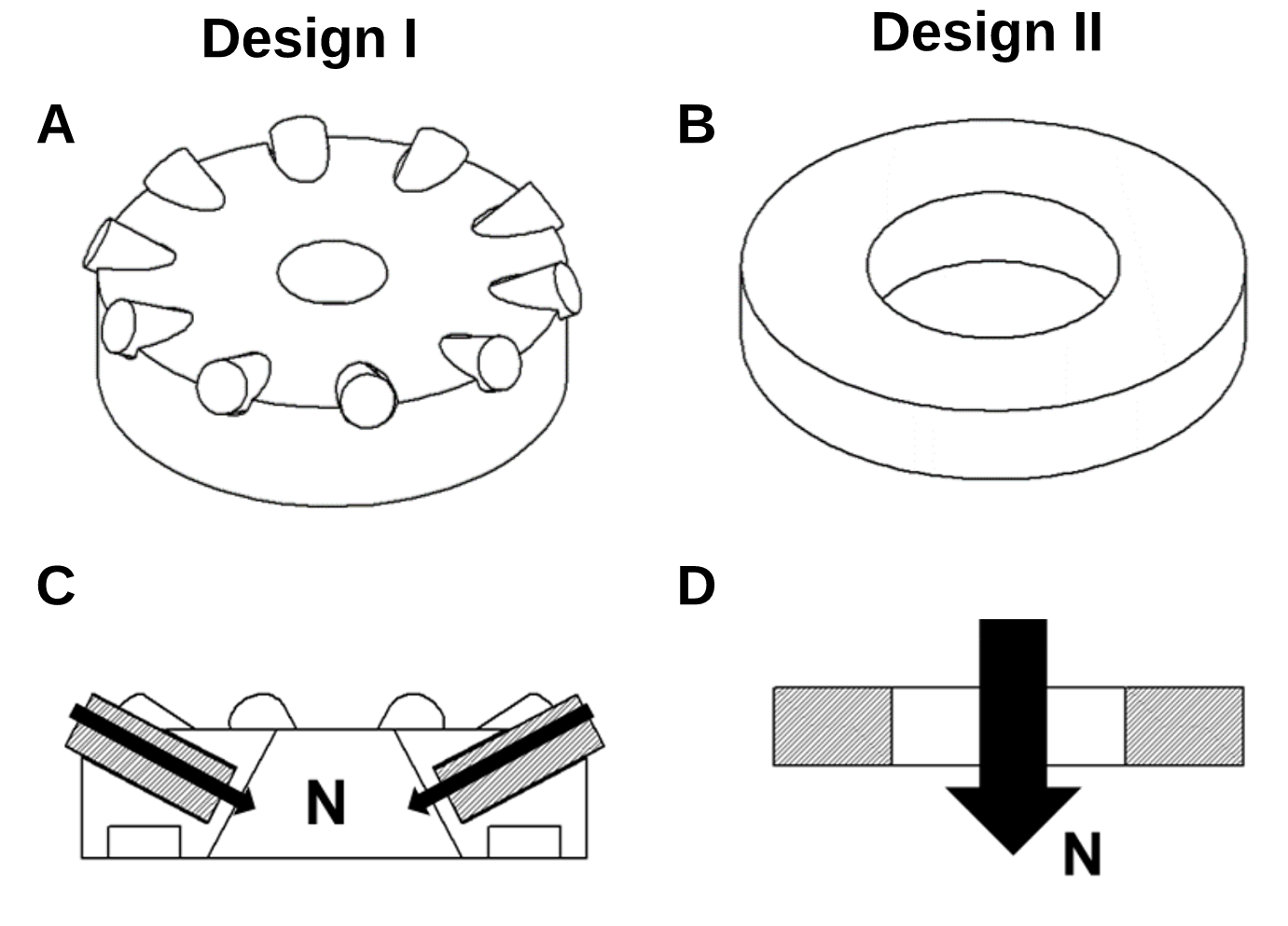


**Supplementary Figure 3. Schematics of Ring Magnets.** Overview **(A, B)** and cross-sectional **(C, D)** views for two ring magnet designs used. **(A, C)** Design I: A 3D-printed ring magnet holder housing 10 cylindrical magnets. **(B, D)** Design II: An axially polarized ring magnet. Arrows denote overall magnetic field direction towards the north pole (N).


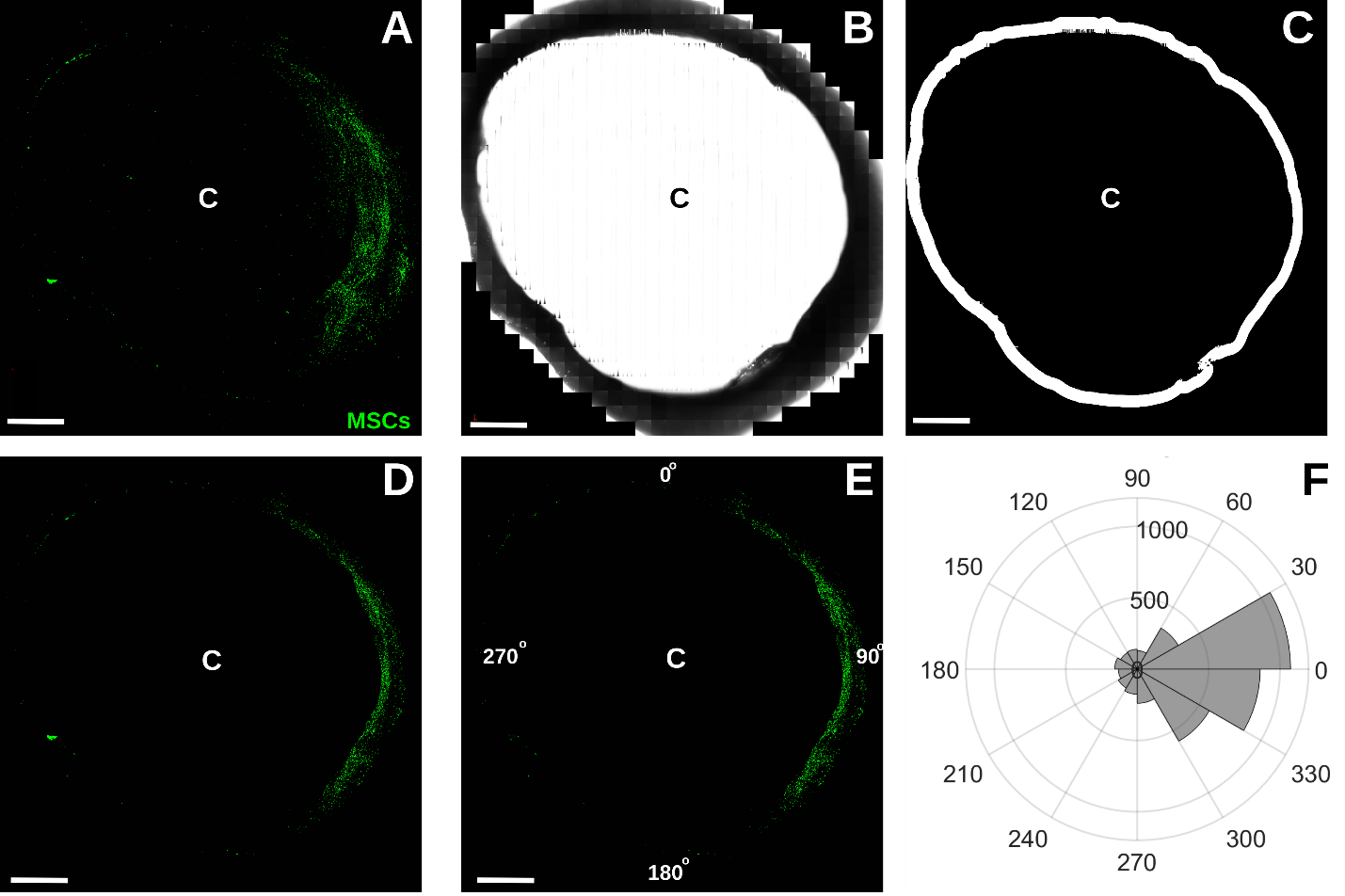


**Supplementary Figure 4. Example of Image Processing for *en face* Micrographs. (A)** Raw fluorescent *en face* micrograph showing CFSE-labeled MSCs in green. “C” denotes the approximate center of the cornea. **(B)** Brightfield *en face* image used for helping to determine the TM location. **(C)** A 1-mm wide ring around the corneal margin was used to create a mask for the TM. **(D)** Example of a masked image showing CFSE-labeled MSC signal within the putative TM. **(E)** Fluorescent debris larger than 100µm was excluded and **(F)** polar histograms were created, showing CFSE-labeled MSC signal (radial coordinate, arbitrary units) within the TM as a function of circumferential location. Scale bars denote 2mm.
